# CanDiD: A Framework for Linking Executive Function and Education

**DOI:** 10.3389/fpsyg.2017.01187

**Published:** 2017-07-13

**Authors:** Niki H. Kamkar, J. B. Morton

**Affiliations:** Department of Psychology, University of Western Ontario, London ON, Canada

**Keywords:** executive function, education, dynamics, development, individual differences, CanDiD

## Abstract

The close association between executive functions (EFs) and educational achievement has led to the idea that targeted EF training might facilitate learning and goal-directed behavior in the classroom. The evidence that training interventions have long-lasting and transferable effects is however decidedly mixed (Melby-Lervåg and Hulme, 2013; Simons et al., 2016). The goal of the current paper is to propose a new CanDiD framework for re-thinking EF and its links to education. Based on findings from basic EF research, the proposed CanDiD framework highlights dynamic and contextual influences on EF and emphasizes the importance of development and individual differences for understanding these effects. Implications for remedial interventions and curriculum design are discussed.

Executive functions (EFs) are a set of processes that are critical for organizing thought and behavior in the service of achieving goals. Although there is no consensus on the specific processes that comprise the “set,” there is general agreement that:

(1) EF makes thinking intelligent by lending stability, foresight, and flexibility to intellectual activity of all kinds;
(2) EF helps solve intellectual problems that have no *a priori* solutions (see Zelazo et al., 1997).

Therefore, thoughts and actions governed by EF can be distinguished from habits, or crystalized forms of mental activity that are acquired gradually through repeated practice and that provide fixed automatic solutions to well-defined problems.

Understanding, the underlying causes of EF development remains a fundamental challenge. One influential position highlights the importance of experience by characterizing the development of EF as a form of skill-learning (Klingberg, 2014). On this view, everyday experience provides opportunities to maintain small amounts of information, filter out salient distractors, and examine situations from multiple vantage points. These experiences are important as they provide children with opportunities to exercise nascent executive skills, and drive functional and anatomical re-organization of associated brain networks. Over time, cognitive and neurophysiological mechanisms underlying EF become more practiced, and by extension, increasingly adult-like. Consistent with this account, targeted practice of working-memory (Klingberg et al., 2002) and task-switching (Karbach and Kray, 2009) paradigms beget measureable changes in cognitive and neurophysiological measures of higher-order cognition (Olesen et al., 2004).

## EF and Education

To the extent that EF lends intelligence to thinking, there has been a long-standing interest in the connection between EF and education. Do individual differences in EF predict success in educational contexts? If so, why? And can interventions that target EF facilitate learning and behavior in the classroom? What we have learned to date is that there is a close connection between EF and achievement in academic settings. Although the reasons are manifold, one is that EF is critical for learning (Bull et al., 2008; Clark et al., 2010). Acquiring new skills in the classroom has much to do with how students organize, seek, and evaluate information, aspects of thinking that depend of EF. EF is also important for managing challenges, be they purely intellectual or socio-emotional in nature. For example, EF predicts not only SAT scores, but also a capacity to cope with stress, uncertainty, and conflict (Mischel et al., 1989).

One implication is that interventions that target EF can facilitate focused behavior in the classroom, especially among students prone to distraction. Of available approaches, working-memory training, in which participants mentally maintain and manipulate small amounts of information over a short delay, is perhaps the most widely recognized. The general approach involves assigning a child a daily regimen of computer-based tasks that demand short-term maintenance and manipulation of small amounts of information. As proficiency improves, the tasks become incrementally more difficult. On some accounts, working-memory training is highly effective not only at remediating EF-related problems, such as short-term memory difficulties among children with ADHD (Klingberg et al., 2002, 2005), but also in promoting “general cognitive enhancement” (Morrison and Chein, 2011), evident in abilities beyond those specifically practiced (Holmes et al., 2009; Bergman-Nutley and Klingberg, 2014).

### Summary and Challenges

One challenge is that evidence for the effectiveness of “EF-training” programs is highly inconsistent. Evaluating working-memory training focuses on the issue of far-transfer effects, namely evidence that training working-memory generalizes to tasks that are different from the task trained on. While evidence of far-transfer effects is arguably the most important for evaluating the utility of working-memory training for use in educational contexts, it also proves to be the least reliable (Melby-Lervåg and Hulme, 2013; Simons et al., 2016). For example, a meta-analysis revealed that there is no convincing evidence that training on working-memory would generalize to other skills including inhibitory control (Melby-Lervåg and Hulme, 2013). More recently, Simons et al. (2016) reported that while there is a body of evidence in support of brain-training interventions improving performance on the trained tasks, there is little evidence of far-transfer to distantly related tasks or everyday cognitive performance (Simons et al., 2016). Indeed, these recent reviews call attention to the weakness in available data, and draw dim conclusions regarding the utility of targeted working-memory training (Melby-Lervåg and Hulme, 2013; Simons et al., 2016).

## CanDiD: An Alternative Framework for Linking EF and Education

In light of this, we propose a new framework for considering links between EF and the classroom. Termed CanDiD, the framework emphasizes Contextual and Dynamic aspects of EF (CanD), and the importance of Development and Individual Differences (DiD). It is based on three assumptions. First, EF is dynamic and subject to contextual influences. Second, development is more than practice, insofar as development constrains the emergent dynamics and contextual influences governing EF. And third, individual differences are fundamental to EF. These assumptions are based on cognitive and neurophysiological studies of EF and its development and have unique implications for thinking about the relationship between EF and the classroom.

### Contextual and Dynamic Aspects of EF

Underemphasized in most cognitive and neurophysiological models is the fact that EF is by its very nature dynamic. Interference suppression, working-memory, and mental flexibility are all subject to a variety of intrinsic (i.e., internal to the child) and extrinsic (i.e., external to the child) influences that lead to continuous and patterned change in the efficacy of these processes over short periods of time. Even cortical networks putatively linked to EF dynamically vary over short timescales, with the nature and complexity of this variability intrinsic to the function of these networks (Hutchison and Morton, 2015; Medaglia et al., 2015; Nomi et al., 2016). Indeed, dynamic variation appears to be a fundamental characteristic of brain function that constrains even elementary aspects of behavior and cognition (McIntosh et al., 2008; Busch et al., 2009; Kucyi and Davis, 2014).

Intrinsic influences that lead to dynamic variability in the efficacy of EF include the body’s natural circadian rhythm. The circadian rhythm is an evolutionarily ancient 24-h cycle of arousal governed by a neuroendocrine circadian clock. Although endogenous, or self-regulating, the circadian rhythm is entrained to the external world through the influence of external cues including light and temperature. Diurnal variations in arousal linked to the circadian rhythm impact EF (Hahn et al., 2012). These effects appear to be specific to effortful forms of cognition such as EF. Indeed, explicit – or effortful – forms of memory retrieval operate best during optimal times of an individual’s circadian cycle, whereas implicit – or effortless – forms of memory retrieval operate best during non-optimal periods of an individual’s circadian cycle. Taken together, these findings point to endogenously governed dynamic changes in thinking styles that evolve over a 24-h period, with effortful and automatic forms of thinking predominating during “optimal” and “non-optimal” circadian periods respectively.

Extrinsic influences on EF are manifold, and contribute to dynamic variation in the efficacy of EF that play out on multiple time scales. One example, referred to as the Gratton effect (Gratton et al., 1992), is driven by the statistics of a task environment, such that tasks saturated with incongruent stimuli show smaller interference effects than do tasks saturated with congruent trials. These effects can be highly localized in time such that the magnitude of an interference effect is markedly attenuated following a single incongruent trial relative to when the same interference effect is measured following a single congruent trial. Varying task contexts are associated with distinct profiles of activity in the brain (Wilk et al., 2012), underscoring the idea that neurocognitive processes that manage conflict are not isomorphic, but subject to dynamic and contextual variability.

Other extrinsic influences that lead to dynamic variations in the efficacy of EF include stress and sleep. Acute stress causes a shift in an organism’s learning style, away from an effortful construction of an allocentric model of the world toward an automatic reward-driven shaping of behavior (Shafiei et al., 2012). For reasons that are not well-understood, sleep duration and quality are linked to the efficacy of EF, with these associations potentially stronger in children than adults (for review, see Turnbull et al., 2013).

In summary, EF is by its very nature dynamic. Core processes, be they cognitively or neurophysiologically conceived, are subject to a variety of intrinsic and extrinsic influences that lead to change in the nature and efficacy of these processes over short periods of time.

### Development: Dynamic and Contextual Constrains

The proposed CanDiD framework assumes that development qualitatively transforms the cognitive, neurophysiological, and neuroanatomical foundations of EF, and therefore at any point in time, fundamentally constrains the dynamical nature of, and contextual influences on, EF.

As one illustration, consider dynamic variation in cortical networks putatively linked to EF. Dynamic variations in cortical network connectivity are an emergent property of highly connected and highly interactive systems such as the brain. Key structural properties of the brain, including path length (i.e., the distance traveled by signals in the brain), conduction velocity (i.e., how rapidly signals travel along pathways in the brain), and signal integrity (i.e., the signal to noise ratio) constrain emergent dynamics (Deco et al., 2011), but also change with development owing to changes in brain size (affecting path length), white matter myelination (affecting conduction velocity), and neurotransmitter availability and receptor density (affecting signal to noise ratio). Thus, development constrains emergent brain dynamics, with potential consequences for the efficacy of EF (McIntosh et al., 2008; Dajani and Uddin, 2015; Medaglia et al., 2015; Hutchison and Morton, 2016; Marusak et al., 2017).

In a similar vein, development constrains how contextual factors impact EF. Throughout development, there are profound changes in sleep duration, onset, and architecture owing in part to changes in the circadian regulation of the sleep-wake cycle. Consequently, optimal periods of the day for effortful goal-directed cognition can be quite different for toddlers, children, and adolescents. Similarly, the proximal and long-term effects of sleep restriction on EF also likely differ for toddlers, children, and adolescents (Bernier et al., 2010; Turnbull et al., 2013). Even the contextual modulation of working-memory and conflict processing efficacy are constrained by development. Whereas older participants retain information about prior processing context and carry this information forward in anticipation of forthcoming cognitive challenges, younger participants treat individual trials as separate instances. Age-related differences in the dynamic adaptation of EF is not only evident in behavior (Chatham et al., 2009), but also in patterns of evoked brain activity (Waxer and Morton, 2011; Wilk and Morton, 2012).

In summary, while the importance of experience for the development of EF is undeniable, it is also the case that development constrains how contextual factors impact EF. Furthermore, age-related differences in EF are likely not reducible to differences in practice, as cognitive, neurophysiological, and neuroanatomical foundations of EF are subject to qualitative transformation over time. Therefore, the CanDiD framework emphasizes the importance of development for understanding contextual influences on emerging EF.

### Individual Differences

Despite the evidence demonstrating typical age-related changes in EF (De Luca et al., 2003), there are substantial inter-individual differences in EF at all developmental stages. The proposed CanDiD model assumes inter-individual differences are a central characteristic of EF that are not reducible to variation between good and poor EF, but reflect a diversity of strategies or approaches to organizing goal-directed behavior and cognition.

As a cognitive trait, EF varies from individual to individual as a consequence of both environmental and genetic factors. Aspects of the early environment such as parental sensitivity (Bernier et al., 2010; Blair et al., 2011, 2014; Hammond et al., 2012), and exposure to adversity (Kamkar et al., 2017) impact EF at a population level by influencing mean EF scores of large groups. At the same time, individual variation around the population mean can be largely accounted for by genetic difference between individuals, given that EF and associated networks are highly heritable (Polderman et al., 2007; Friedman et al., 2008; Lenroot et al., 2009; Anokhin et al., 2011; Miyake and Friedman, 2012).

The close connection between environmental and genetic influences suggests variation in EF does not follow a continuum of good to poor, but reflects a principled relationship between EF and the nature of a child’s early environment. One example is gene-environment correlation, whereby individuals select environments that match their own genetic propensities (Scarr and McCartney, 1983; Plomin and Deary, 2015). This is best reflected in age-related increases in heritability estimates of EF and related constructs like intelligence (Deary et al., 2009, 2010, 2012; Haworth et al., 2010; Tucker-Drob et al., 2013; Tucker-Drob and Briley, 2014; Plomin and Deary, 2015; Plomin et al., 2016). Another example is gene-environment interactions in which certain genetic variants bestow phenotypic stability while others bestow phenotypic plasticity (Bennett et al., 2002; Belsky and Pluess, 2009). Gene-environment interactions are evident in selected aspects of EF such as self-regulation and decision-making (Carré et al., 2012).

Taken together then, there is evidence that diversity is not only the starting point of development, but is also evident in developmental trajectories. Young children differ in the way they strategically organize their thoughts and actions, and will consequently seek out environments that complement their preferred approach to self-regulation as they grow older. In light of this important inter-individual variability, the CanDiD framework emphasizes differences between children in terms of the development of EF.

## Speaking “CanDiD-ly” About EF and Education

### CanD: Context and Dynamics

Recognizing the contextual and dynamic nature of EF casts a new light on the relationship between EF and the classroom. Re-thinking this relationship has implications for how we understand and manage student behavior in educational settings. Consider, as an example, inattentiveness and distractibility in the classroom. If we approach the analysis of this style of thinking from the standpoint of EF as a stable cognitive trait that can be trained through targeted practice, we isolate this style of thinking from the context in which it evolves and overlook important intra-individual variability in intellectual focus that might serve as a critical building-block for remediation. Thinking “CanDiD-ly” on the other hand, shifts priorities toward cataloging potential contextual influences on EF and identifying variability in attentiveness over time. For instance, is the child’s inattentiveness and distractibility related to unhealthy sleep routines? Is the child acutely (or chronically) stressed, either at home or amongst their peers? Is the child more attentive at certain times of the day than others? Thinking “CanDiD-ly” about inattentiveness gives priority to contextual influences on and dynamic variation in EF-related behavior. It also underscores the importance of working closely with students and parents to identify factors that influence a child’s ability to concentrate, or times of the day when a student’s focus and readiness to learn is optimal. Consistent with the spirit of this suggestion is the American Academy of Pediatrics recommendation that middle and high schools start at 8:30 am or later so that students can obtain the 8.5–9.5 h of sleep they require (Adolescent Sleep Working Group, 2014). Implicit in this approach is the notion that variation in inattentiveness can be part of larger cycle of arousal (diurnal or otherwise). Thinking “CanDiD-ly,” the priority becomes adjusting the child’s environment or daily routine to maximize the likelihood that the instructor is teaching when the children are ready to learn.

### DiD: Development and Individual Differences

Thinking “CanDiD-ly” about EF, we need to recognize there are qualitatively different styles of learning that are deeply rooted in the nature of individual children. One implication is a need to move from passive modes of instruction, to active modes in which children are granted more active roles in selecting, modifying, and creating their own educational experiences. On this view, an “equal” educational system is not one in which all children are exposed to exactly the same environments, but one in which all children are given an opportunity to select learning environments that accommodate their learning strengths (Asbury and Plomin, 2013).

Furthermore, rather than using computerized tasks that train a narrow range of cognitive processes, programs that allow for broad practice in EF-promoting activities may be more successful. Aerobic exercise, pretend play, yoga, and mindfulness meditation have all been implicated in improving EF (Diamond and Lee, 2011). A curriculum that targets broad activities and has shown promise in improving EF is the *Tools of the Mind (Tools)* curriculum, which constitutes activities including pretend play, self-regulatory private speech, and dramatic arts. These activities are said to promote EF because they require inhibitory control. For example, in dramatic arts, children must inhibit acting out of character (Diamond and Lee, 2011). When compared against the District’s version of Balanced Literacy curriculum (dBL), participants in the *Tools* curriculum significantly outperformed those in the dBL curriculum on measures of inhibitory control (Diamond et al., 2007). *Tools* differs from the working-memory training discussed previously because it allows for practice in a broad range of EF-promoting activities, rather than training on a specific task and expecting gains on a construct as broad as EF; thus, *Tools* may not suffer of as many issues related to far-transfer effects.

## Concluding Remarks

The present paper offers a new framework for thinking about EF and its links with education, one that is informed by basic research into the nature of EF and its development, and departs from more conventional approaches to these issues. With this framework, researchers are at liberty to conduct studies that assess what contextual and dynamic factors might constrain EF, and how individual differences at the genetic and environmental levels might be related to the development of EF. In the context of education, using a CanDiD approach may allow teachers to take note of these contextual, dynamic, and individual factors and to use this knowledge in tailoring an educational curriculum that considers the needs of the child rather than expecting children to adjust to a one-size-fits-all education system.

## Author Contributions

All authors listed have made a substantial, direct and intellectual contribution to the work, and approved it for publication.

## Conflict of Interest Statement

The authors declare that the research was conducted in the absence of any commercial or financial relationships that could be construed as a potential conflict of interest.
